# Determining the Effects of Transcranial Direct Current Stimulation on Tinnitus, Depression, and Anxiety: A Systematic Review

**DOI:** 10.3390/brainsci12040484

**Published:** 2022-04-08

**Authors:** Bas Labree, Derek J. Hoare, Lauren E. Gascoyne, Polly Scutt, Cinzia Del Giovane, Magdalena Sereda

**Affiliations:** 1 NIHR Nottingham Biomedical Research Centre, Ropewalk House, 113 The Ropewalk, Nottingham NG1 5DU, UK; derek.hoare@nottingham.ac.uk (D.J.H.); polly.scutt@nottingham.ac.uk (P.S.); magdalena.sereda@nottingham.ac.uk (M.S.); 2 Hearing Sciences, Mental Health and Clinical Neurosciences, School of Medicine, University of Nottingham, Nottingham NG7 2UH, UK; 3 Sir Peter Mansfield Imaging Centre, School of Physics and Astronomy, University of Nottingham, Nottingham NG7 2XQ, UK; lauren.gascoyne@nottingham.ac.uk; 4 Department of Medical and Surgical Sciences for Children and Adults, University-Hospital of Modena and Reggio Emilia, 41124 Modena, Italy; cinzia.delgiovane@unimore.it; 5 Institute of Primary Health Care (BIHAM), University of Bern, Mittelstrasse 43, 3012 Bern, Switzerland

**Keywords:** transcranial direct current stimulation (tDCS), tinnitus, depression, anxiety, stimulation parameters

## Abstract

(1) Background: Tinnitus is the awareness of a sound in the absence of an external source. It affects around 10–15% of people, a significant proportion of whom also experience symptoms such as depression or anxiety that negatively affect their quality of life. Transcranial direct current stimulation (tDCS) is a technique involving constant low-intensity direct current delivered via scalp electrodes. It is a potential treatment option for tinnitus, as well as tinnitus-related conditions such as depression and anxiety. This systematic review estimates the effects of tDCS on outcomes relevant to tinnitus. In addition, it sheds light on the relationship between stimulation parameters and the effect of tDCS on these outcomes; (2) Methods: Exhaustive searches of electronic databases were conducted. Randomised controlled trials were included if they reported at least one of the following outcomes: tinnitus symptom severity, anxiety, or depression. Where available, data on quality of life, adverse effects, and neurophysiological changes were also reviewed. GRADE was used to assess the certainty in the estimate; (3) Results: Meta-analyses revealed a statistically significant reduction in tinnitus (moderate certainty) and depression (low certainty)-but not anxiety-following active tDCS compared to sham control. Network meta-analyses revealed potential optimal stimulation parameters; (4) Conclusions: The evidence synthesised in this review suggests tDCS has the potential to reduce symptom severity in tinnitus and depression. It further narrows down the number of potentially optimal stimulation parameters.

## 1. Introduction

Tinnitus is the conscious awareness of a tonal or composite noise for which there is no identifiable corresponding external acoustic source [1]. The perceived location of the tinnitus varies, but most often it is perceived in the ear(s) or head. It is estimated that around 15% of adults experience tinnitus. Whether people with tinnitus seek out clinical intervention varies, depending on symptom severity [2].

Current clinical treatment strategies include cognitive behavioural therapy (CBT), education, sound therapy, and combinations thereof such as Tinnitus Retraining Therapy (TRT) [3]. However, the effectiveness of these treatments is variable. Several experimental interventions for tinnitus have been proposed, such as pharmacological interventions [4,5,6,7], dietary supplementation [8] and neuromodulation treatments [9]. One experimental treatment in the last category is transcranial direct current stimulation (tDCS), a neuromodulation technique that involves the application of a weak electrical current (0.5–2 mA) to the cortex via electrodes applied to the scalp. Several different neuromodulation techniques exist, though none are recommended for tinnitus, since little evidence is available for its effectiveness and what evidence there is, is not of a sufficiently high standard to warrant recommendation [10]. Data on tDCS is the least limited and the number of randomised controlled trials published in recent years makes it the most viable candidate for evidence synthesis. The key mechanism involved is the subthreshold modulation of neuronal membrane potentials leading to changes in cortical excitability and neuronal activity [11]. Rather than induce neural activity directly, tDCS is thought to increase or decrease the likelihood of action potentials in a neuronal population, through this change in excitability. Investigators of many disorders which are at least partially underpinned by abnormal neurophysiological states have used tDCS both as a tool to better understand these disorders and as a potential treatment. The treatment rationale is that, if maladaptive neural activity can be inhibited and normal activity restored this may result in an improvement of symptoms, particularly if this leads to lasting neuroplastic changes [12,13]. TDCS has been trialled for many different disorders which are in part explained by maladaptive central nervous system activity, including tinnitus, neuropathic pain with varying aetiologies [14,15], mood disorders [16,17,18], aphasia and other pathologies [19].

A particular association exists between tinnitus and mood disorders. A recent systematic review found a median prevalence of depression of 33% in adults with tinnitus (interquartile range 19–49%) [20]. A population study with a large sample (*n* = 21.4 million respondents with tinnitus) which sought to quantify the relationship between tinnitus, depression and anxiety found further evidence for this relationship. In that study, 26.1% of participants who had tinnitus but only 9.2% of controls reported symptoms of anxiety in the previous 12 months. A strikingly similar pattern was observed for depression; 25.6% of participants with tinnitus but only 9.1% of controls reported symptoms of depression in the previous 12 months. Furthermore, those reporting tinnitus as a “big” or “very big” problem were more likely to report anxiety and/or depression [21]. While tempting, it cannot be assumed that this increased prevalence of symptoms of depression and anxiety in this population is the result of tinnitus. The evidence currently available on the relationships between tinnitus, depression and anxiety is robust but correlational. With causal evidence lacking, it is important to refrain from assumptions regarding the direction of this relationship. In the absence of a clear mechanism by which tinnitus and depression interact Geocze and colleagues [22] proposed three possible associations: (1) depression affecting tinnitus, (2) tinnitus predisposing its sufferers to depression, and (3) tinnitus appearing as a comorbidity with depression. Despite the lack of clarity on the mechanism of interaction between tinnitus and co-occurring depression or anxiety, this association is not only observed in observational data. Interventional studies trialling tDCS in a sample of people with tinnitus have found that improvements in tinnitus symptom severity were accompanied by improvements in depression [23,24].

There is currently no consensus on the effectiveness of tDCS for tinnitus. One recent systematic review found a greater reduction in tinnitus-related distress for groups treated with tDCS compared to controls [25]. However, another recent systematic review found no therapeutic effect of tDCS on tinnitus [26]. One factor that complicates the synthesis of existing evidence in this area is the heterogeneity of treatment protocols. Across studies of tDCS for tinnitus, stimulation parameters such as electrode montage, current intensity, duration, and frequency of stimulation sessions vary widely. Currently there is no consensus on the optimal setup for improvements in tinnitus symptom severity. Therefore, when trials in this area report a null effect, it is difficult to know whether this is due to tDCS being ineffective for tinnitus, or due to the stimulation parameters employed being suboptimal.

This systematic review assessed the effects of tDCS on tinnitus symptom severity, depression, and anxiety. Where reported we also assessed adverse effects and the effects of tDCS on quality of life and neurophysiological change. The relationships between stimulation parameters and these effects were also reviewed.

## 2. Materials and Methods

The protocol for this review was designed and reported according to the PRISMA-P [27] and has been registered on PROSPERO (registration number: CRD42020185567). The full protocol, including an example of the full search strategy has been published previously [28].

Databases and search strategy: Electronic searches for peer-reviewed journal articles were performed in the Cochrane Register of Studies online (the Cochrane Ear, Nose and Throat Disorders Group Register and CENTRAL, current issue); PubMed; EMBASE; CINAHL; LILACS; KoreaMed; IndMed; PakMediNet; CNKI; AMED; PsycINFO; Web of Science; ClinicalTrials.gov; ICTRP and Google Scholar using the following search terms: transcranial Direct Current Stimulation OR tDCS AND tinnitus OR depression OR anxiety OR quality of life OR adverse effects OR neurophys*.

Eligibility criteria: Published or in press peer-reviewed journal articles reporting randomised controlled trials (RCTs) or cross-over trials (eligible if data from before the crossover could be extracted, to avoid the potential for a carry-over phenomenon) were included. Only records available in English were included and no date restrictions were applied. Records were included if they reported an RCT with a sample that comprised participants aged 18 years or over who had been diagnosed with any health condition and received at least one session of tDCS using any electrode montage and any stimulation parameters. Studies where the intervention was high definition tDCS were excluded. Studies had to include a control arm which consisted of either sham (placebo) tDCS, no intervention, or a waiting list. Records had to include data on at least one of the primary outcomes of this review-tinnitus, depression, anxiety. Where available, data on quality of life, adverse effects, and neurophysiological change were also included.

Screening, data extraction and quality assessment: Record screening, data extraction, and risk of bias assessments were conducted independently by two authors, following which consensus was achieved by discussion between the authors in question. If not reported or provided by the authors, standard deviations were estimated using the available data such as standard errors, confidence intervals, p and t values. Where data were only available in graph form, authors approximated numerical data using semi-automatic software developed for this purpose (https://automeris.io/WebPlotDigitizer/; accessed on 14 January 2022). Risk of bias assessment was conducted as guided by the Cochrane Handbook for Systematic Reviews of Interventions [29]. No studies were excluded based on their risk of bias rating.

Descriptive and meta-analyses: Data synthesis was performed using RevMan 5.4.1. [30]. Outcomes were analysed separately. If data could not be obtained, studies were excluded from the analysis. Meta-analyses were performed wherever multiple studies reported a given outcome and combining studies was methodologically and statistically appropriate. For those studies that were not suited to this approach, a narrative synthesis was conducted. Data from RCTs were pooled using random-effects models, given the heterogeneity of aggregated effect sizes found. This was calculated using Cochran’s Q statistic (χ^2^ test with K-1 degrees of freedom, K being the number of studies) and the I2 statistic (with percentages of approximately 25%, 50% and 75% of I2 being interpreted as low, medium, and high heterogeneity respectively [31]. By this definition, heterogeneity was medium for tinnitus and high for depression and anxiety. Dichotomous data were pooled using the RR measure. Continuous tinnitus data were pooled using the mean difference because all studied that included tinnitus as an outcome measure reported Tinnitus Handicap Inventory (THI) scores. Since multiple different instruments were used in studies reporting depression and anxiety, standardised mean difference was used. The psychometric properties of questionnaires were considered to judge their suitability for pooling. Data were only included from multi-item questionnaires that show similar responsiveness and could be assumed to measure the same underlying construct (high convergent validity) as other multi-item questionnaires for the same outcome. We used the GRADE framework to assess the certainty in the evidence [32]. Where there were sufficient studies within meta-analyses, funnel plots were produced and inspected to consider risk of bias.

For outcomes with sufficient data present, network meta-analyses were conducted using the network package in Stata [33], to clarify the effects of differences in stimulation parameters (electrode montage, current intensity, stimulation duration and number of stimulation sessions). A separate network meta-analysis was conducted for each of these parameters.

## 3. Results

### 3.1. Study Selection

**Figure 1:**
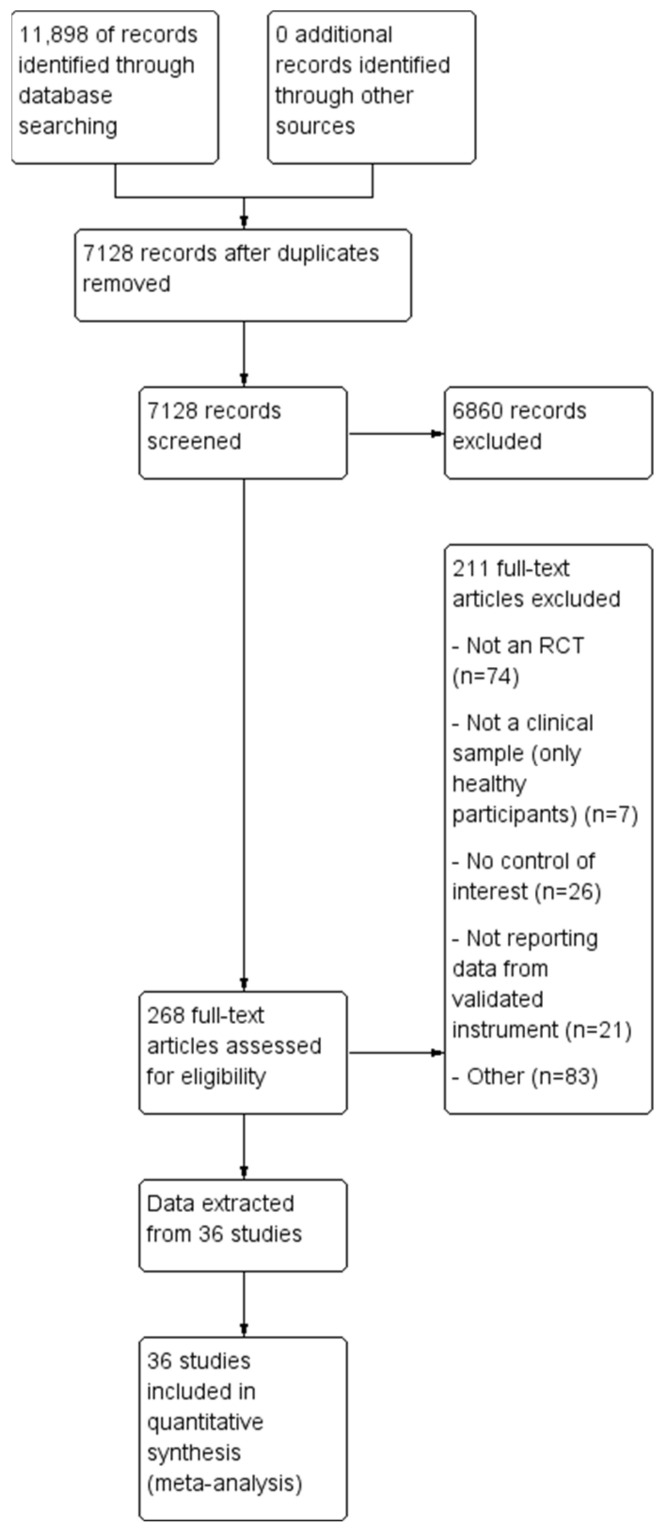
Study selection diagram.

For an overview of the study selection process see Figure 1. Systematic searches were conducted on 22 June 2020 with a final update search conducted on 30 November 2021. Searches yielded 11,898 records. Duplicates were removed, followed by screening for inclusion based on title and abstract. For the remaining records full texts were retrieved and assessed for inclusion. Data from 36 records were included in the review and all 36 were included in meta-analyses.

### 3.2. Study Characteristics

Characteristics of each included study are summarised in Table 1. 35 parallel RCTs and one crossover RCT where data from before the cross were available were included. All included studies included a sham condition (as opposed to waiting list control/no intervention). Six studies with a total of 188 participants reported tinnitus as an outcome, 30 studies with a total of 1206 participants reported depression as an outcome, and 15 studies with a total of 592 participants reported anxiety as an outcome. The included studies covered a wide range of conditions. While there was variability in stimulation parameters, some patterns were observed. For instance, a current intensity of 2 mA was used in nearly all studies (30 out of 36). Electrode montages varied the most across studies, although some electrode placements occurred frequently enough to allow for further analysis.

### 3.3. Risk of Bias

**Figure 2:**
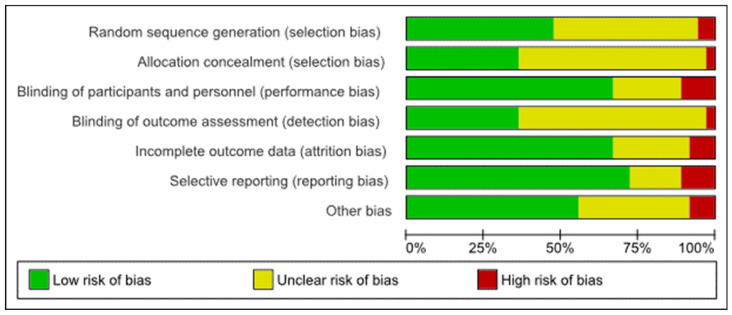
Risk of bias graph.

Risk of bias scores are summarised in Figure 2. For a breakdown of risk of bias scores for each study, see Supplementary Table S1. Risk of selection bias due to inadequate random sequence generation was rated as high for two studies [34,51] and high due to inadequate allocation concealment for one study [51]. Risk of performance bias was judged to be high due to inadequate blinding of participants and personnel in four studies [36,54,55,59]. Risk of detection bias was held to be high due to inadequate blinding of outcome assessments in one study [55]. Risk of attrition bias due to incomplete outcome data was found to be high in three studies [24,57,65]. High risk of reporting bias due to selective reporting was found in four studies [53,55,60,62]. Risk of bias from other sources was found in three studies [40,55,62].

### 3.4. Results of Syntheses

#### 3.4.1. Primary Outcomes

**Figure 3:**
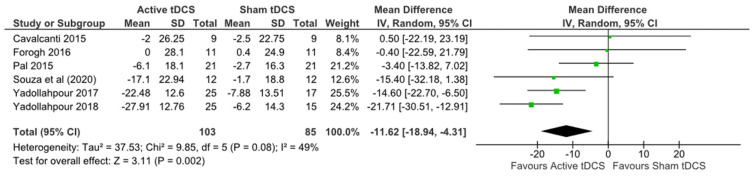
Forest plot tinnitus.

Meta-analyses were conducted for each of the primary outcomes-tinnitus, depression, and anxiety. Random effects models were used, due to the heterogeneity encountered. A statistically significant effect favouring active tDCS over sham was observed for tinnitus (MD −11.62, 95% CI −18.94, −4.31), moderate certainty (Table 2), heterogeneity: Tau2 = 37.53; Chi2 = 9.95, df 5 (*p* = 0.08); I2 = 49% (Figure 3).

**Figure 4:**
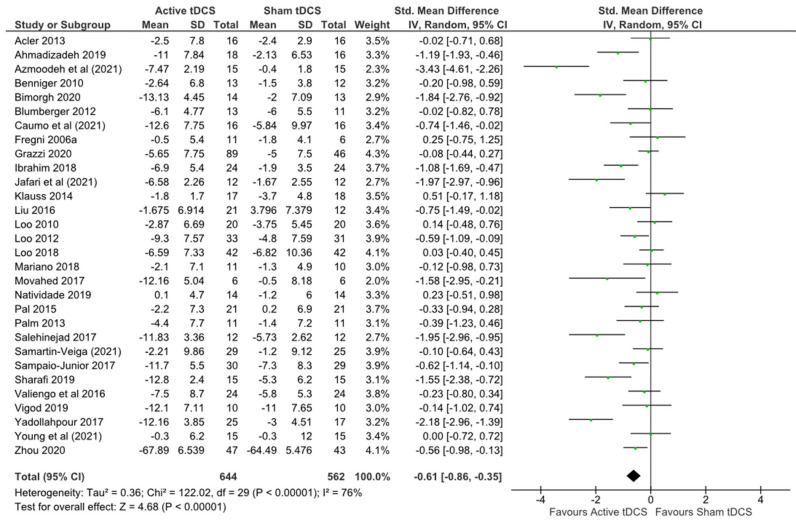
Forest plot depression.

A statistically significant effect favouring active tDCS over sham was also observed for depression (SMD −0.61, 95% CI −0.86, −0.35), low certainty (Table 3) heterogeneity: Tau2 = 0.36; Chi2 = 122.02, df 29 (*p* = 0.00001); I2 = 76% (Figure 4).

**Figure 5:**
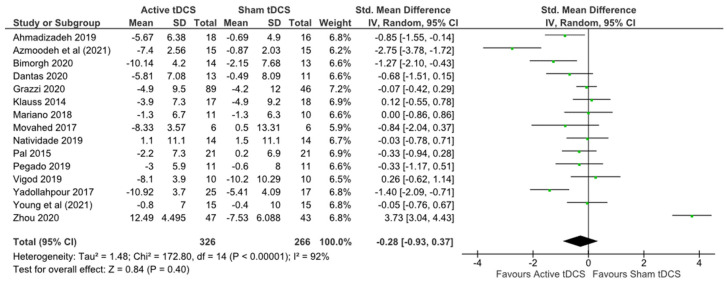
Forest plot anxiety.

No statistically significant effect was observed for anxiety (SMD −0.14, 95% CI −0.31, 0.04), very low certainty (Table 4) heterogeneity: Tau2 = 1.48; Chi2 = 172.80, df 14 (*p* < 0.00001); I2 = 95% (Figure 5).

**Figure 6:**
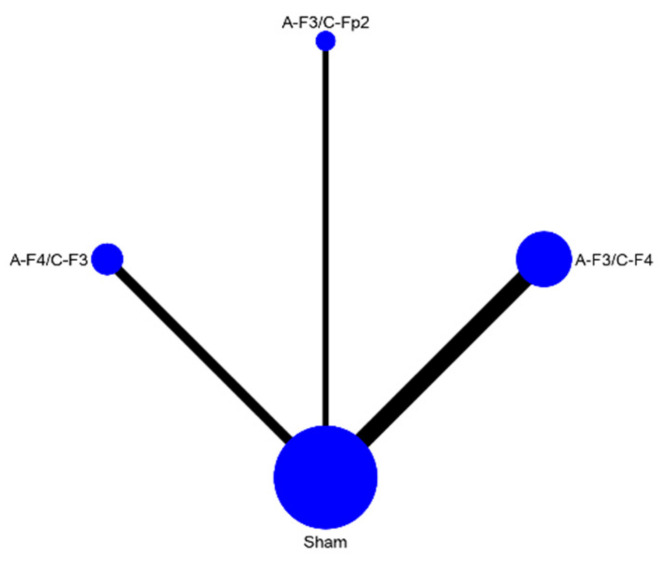
Network map electrode montage.

**Figure 7:**
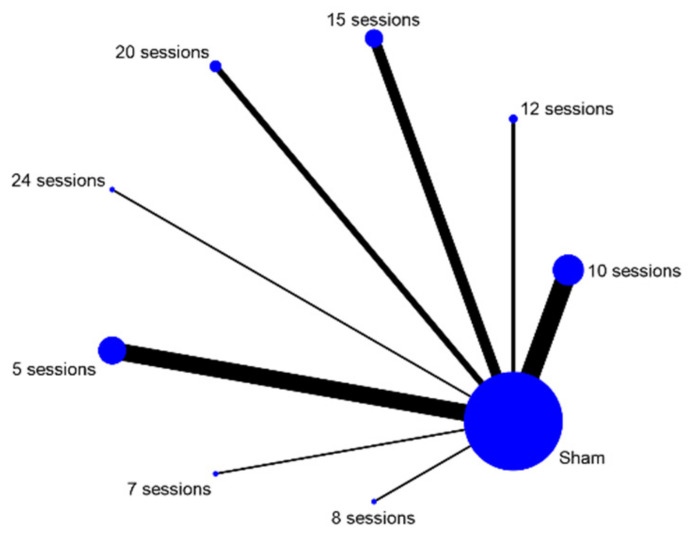
Network map number of sessions.

**Figure 8:**
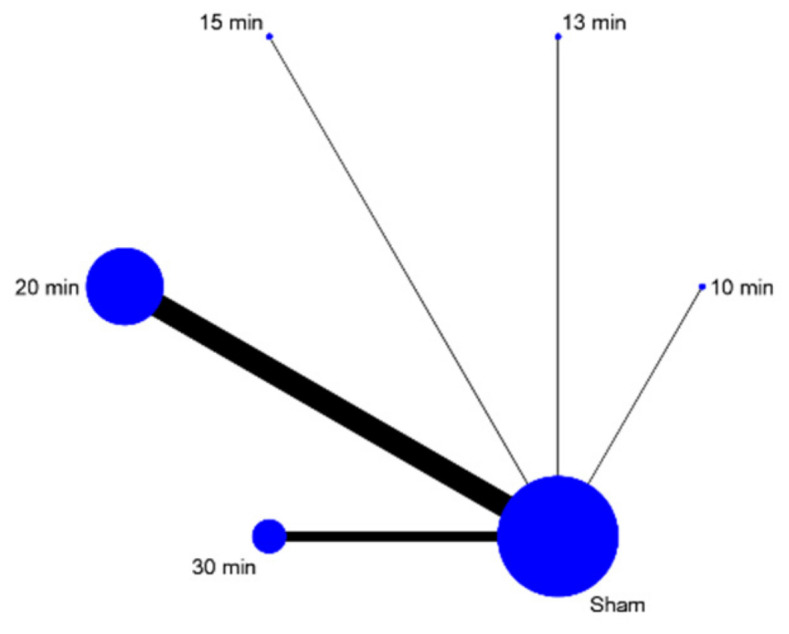
Network map session duration.

The quantity of the data on tinnitus symptom severity was too limited for network meta-analysis. As a result, no disentanglement by tDCS parameters took place. The data extracted for depression were sufficient in quantity to allow for further analysis by three stimulation parameters: electrode montage, duration of stimulation session, and number of sessions (Figure 6, Figure 7 and Figure 8). Since nearly all included studies used the same current intensity, no network meta-analysis was conducted for this parameter. The network meta-analyses provided a ranked list of parameters in each category, with the estimated probability that each setting for that parameter is the best and Monte Carlo standard error estimations (Table 2, Table 3 and Table 4).

Each network meta-analysis showed that sham was the least probable to be effective.

Sufficient data for grouping in the network was present for three electrode montages: the anode electrode over F3 and the cathode over F4 (A-F3/C-F4), the anode electrode over F4 and the cathode over F3 (A-F4/C-F3) and the anode over F3 and the cathode over Fp2 (A-F3/C-Fp2). A-F3/C-F4 and A-F3/C-Fp2 are nearly equally likely to be optimal, with probabilities of 38.9% and 38% respectively. 

Studies varied widely in the number of tDCS sessions applied. The network meta-analysis showed seven sessions was the most probable to be optimal with a probability of 89.3%. It is worth noting that five, eight, and 10 sessions were assigned high mean ranks (5.5, 6.1 and 2.3 respectively) as well, indicating that the true optimum may be somewhere in between.

The network meta-analysis for session duration showed that 20 min is most probably optimal at 58.6%. Session duration of 30 min has a relatively high mean rank (2.4), again indicating that the true optimum may be more than 20, but less than 30 min. Based on this model, session durations of under 20 min (10, 13 and 15 min) are unlikely to be optimal.

#### 3.4.2. Secondary Outcomes

**Figure 9:**
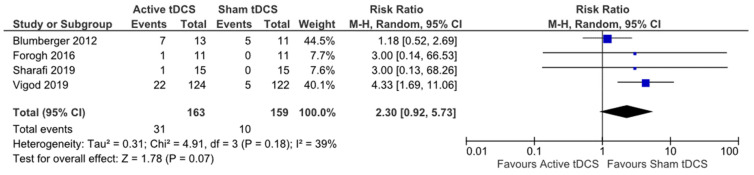
Forest plot adverse effects.

Data from secondary outcomes—adverse effects, quality of life, and neurophysiological change were also extracted if reported in the included studies. Those studies that included a measure of quality of life did not measure and/or report their results for this outcome in a way that allowed for meta-analysis. Adverse effect data were summarised as risk ratios (Figure 9). There was no statistically significant difference in the number of adverse effects following active tDCS compared to sham (RR 2.30, 95% CI 0.92, 5.73).

The included studies varied in how they reported adverse effects. Most studies did not report adverse effects in a way that allowed for inclusion in this analysis. For instance, some reported the overall number of adverse effects, some reported the number of participants who experienced adverse effects, and others the percentage of participants who reported adverse effects. Whether adverse effects were observed in the active tDCS or sham group was often not reported. In total, 5 studies reported observing no adverse effects, 15 studies reported observing no serious adverse effects and 10 did not report any information about adverse effects. The way in which adverse effects were reported makes it difficult to provide an accurate number of adverse effects across all included studies. However, a pattern can be observed in those studies that did report adverse effects. Most adverse effects reported were mild, such as tingling on the scalp or an itching/burning sensation. 

Reporting on adverse effects was not consistent across studies and there seems to be no consistency amongst these studies on what constitutes a mild versus serious adverse effect. Studies that reported mild adverse effects usually reported tingling, itching and/or or a burning sensation on the scalp. However, one study reported that no adverse effects had been observed, but that all of their participants had experienced tingling. Another study reported that there had been no serious adverse effects, despite one participant having withdrawn from the study due to scalp discomfort. Across all included studies, only three participants were reported by authors to have experienced serious adverse effects: two reported worsening of tinnitus (one participant in active and one in sham condition) and one participant suffered first degree burns to their earlobe/mastoid due to an incorrectly positioned electrode. 

Five studies reported data on quality of life, but none did so in a way that allowed for meta-analysis. Acler and colleagues [34] reported no overall score, only subcomponents of their chosen instrument and did not provide means or standard deviations before and after intervention. This study did report the result of an omnibus test, which suggested a greater improvement in quality of life following active tDCS, compared to sham. Benninger et al. [37] similarly reported no overall score and no means or standard deviations before and after intervention. Their statistical testing did not find a significant difference in quality of life between tDCS and sham groups. Klauss et al. [48] only reported the result of statistical testing, which found no significant difference between tDCS and sham groups. Similarly, Liu et al. [49] only reported the results of statistical testing, which did not detect a significant difference in quality of life between active and sham tDCS groups. Loo and colleagues [52] also did not report quality of life data in a way that allowed for meta-analysis but did report the result of statistical tests. They found a significant effect of time, i.e., quality of life scores had improved following the intervention, but there was no statistically significant difference between participants who had received active tDCS and those who received sham.

Only two studies reported a measure of neurophysiological change using EEG. Insufficient data were present for synthesis. Liu et al. [49] reported an increase in delta frequency band power over the frontal region and delta, alpha, and theta band power in the occipital region following tDCS compared to sham, but none of these increases were statistically significant. Souza et al. [62] reported a decrease in theta and beta frequency band power in the inferior temporal gyrus and fusiform gyrus structures following tDCS compared to sham. These decreases were statistically significant in the group of participants that had their eyes closed, but not those who had their eyes open. The authors also observed a significant negative correlation between power in all frequency bands (except for delta) and post-intervention improvements in tinnitus. The authors interpreted this as an indication that higher power in alpha, theta, beta, and gamma frequency bands were associated with greater reductions in tinnitus symptom severity.

### 3.5. Reporting Bias

**Figure 10:**
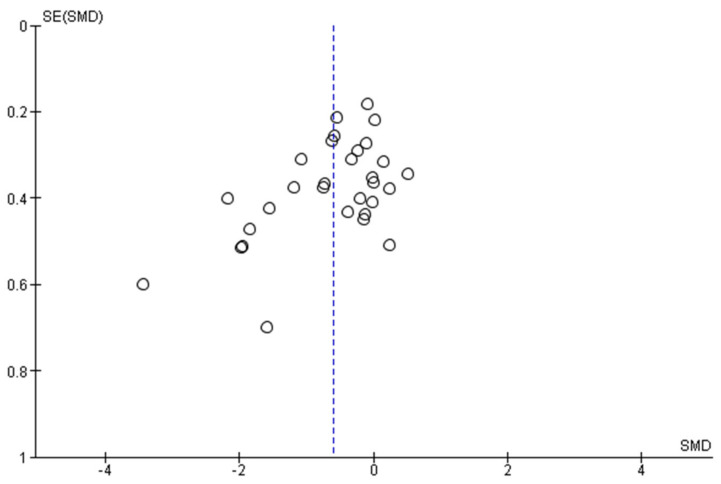
Funnel plot depression.

**Figure 11:**
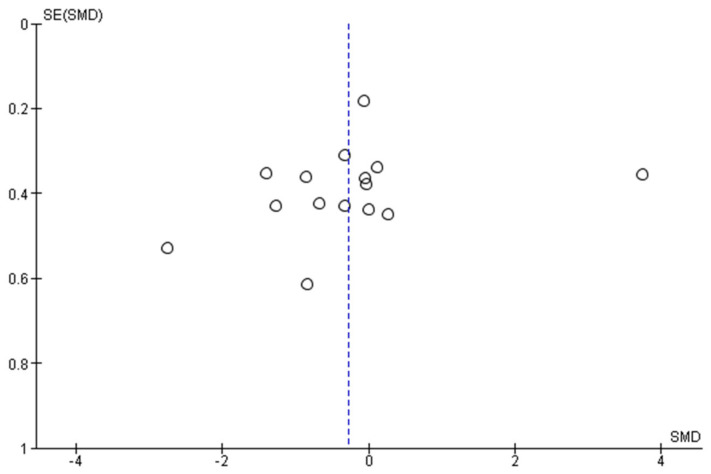
Funnel plot anxiety.

No funnel plot could be generated for studies reporting tinnitus, as these were too few. The funnel plot for depression (Figure 10) shows seven studies that fall clearly outside the 95% confidence intervals and another six that intersect with the 95% confidence interval lines. The funnel plot for anxiety (Figure 11) shows four studies that fall outside the 95% confidence intervals and one that intersects with it. This indicates a considerable amount of asymmetry for both outcomes, potentially indicating publication bias (studies giving significant results were more likely to be published than studies giving nonsignificant results). However, there are other factors that may have contributed to the asymmetries such as differences between studies in methodological quality and/or sample, and true heterogeneity in intervention effects, and artefacts due to sampling variation.

### 3.6. Certainty of Evidence

While we are confident in the accuracy of the effect observed in the meta-analysis of the tinnitus data, it must be noted that only six studies were entered into this analysis. We are similarly confident in the results of the meta-analysis of depression data, given the large, aggregated sample. It is important to point out, however, that this data came from studies covering many different disorders, meaning the cumulative sample is particularly heterogeneous. This heterogeneity is observed alongside a considerably asymmetrical funnel plot, suggesting a degree of selection bias. Considering the association between depression and anxiety, it is perhaps surprising to find a significant effect in favour of tDCS in depression, but not in anxiety. It is difficult to say whether this is because there is truly no effect, or because there is insufficient data available to provide adequate statistical power. The certainty of the evidence from the meta-analyses for the primary outcomes suggests tDCS likely results in a large reduction in tinnitus symptom severity (moderate certainty) and may result in a large reduction in depression (low certainty). The evidence is very uncertain about the effect of tDCS on anxiety. The GRADE assessments are detailed in Table 5, Table 6 and Table 7.

## 4. Discussion

This systematic review assessed the effects of tDCS on tinnitus symptom severity, depression, and anxiety. The relationship between stimulation parameters and these effects were also reviewed. Where reported we also assessed the effects of tDCS on quality of life and neurophysiological change and associated adverse effects.

The meta-analyses show a statistically significant effect in favour of active tDCS for tinnitus as compared to sham. This finding is in line with previous research, showing an improvement in tinnitus symptom severity following active tDCS [25]. However, it must be noted that this result is based on data obtained from 188 participants across five studies, each of which used different but overlapping sets of stimulation parameters. To progress research into the potential clinical application of tDCS for tinnitus, it must be further optimised. To achieve this, consistency in stimulation parameters and outcome reporting will be necessary in future trials.

The meta-analyses further showed a statistically significant effect in favour of active tDCS for depression compared to sham. This is in line with previous research that showed an improvement in depression following active tDCS [17]. The similarity in effects on tinnitus and depression is not surprising, given the observation of improvements in tinnitus and depression in trials of tDCS that measured both [23,24]. That this effect resulted from the synthesis of data from 1206 participants across 30 trials, allows for greater confidence in the result as well as for its disentanglement. The subsequent network meta-analyses shed some light on which stimulation parameters might be most effective. 

Perhaps the most variable parameter in tDCS research is electrode montage. Electrodes can be produced in any shape or size, although 5 × 5 cm^2^ and 7 × 5 cm^2^ square or rectangular electrodes are most common. While most studies used one anode and one cathode electrode, even this can vary. Finally, the options for positioning of the electrodes are almost limitless. Most studies use the EEG 10–20 system for reasons of practicality and reproducibility. However, even within this system there are many different options. Several set-ups are encountered in the literature. Common electrode montages, both in the tinnitus and depression literature, include those that aim to stimulate the dorsolateral prefrontal cortex (dlPFC) or other frontal structures. For the electrode montage network meta-analysis studies using three electrode montages were grouped together: those that placed the anode electrode over F3 and the cathode over F4, those that place the anode over F4 and the cathode over F3 and those that place the anode over F3 and the cathode over Fp2. The network meta-analysis ranks the first and last of these very nearly equally as most likely to be effective. Neither of these is modelled to be overwhelmingly probable to be effective and there is a lot of uncertainty around the estimates. These imprecise results may be due to the limited quantity of existing data and a high degree of heterogeneity among the available data. Nevertheless, there is also a possible anatomical explanation for these findings: both montages send a direct current from the left side of the anterior part of the head to the right side of the anterior part of the head, one somewhat more rostral than the other. Since tDCS (excluding HD-tDCS) has limited spatial precision, the electric field distribution generated by these montages would overlap and likely stimulate many of the same structures. This may mean that, rather than one optimal electrode montage defined by 10–20 coordinates, any montage that creates an electric field of sufficient strength and size in the frontal cortex is likely to be effective. It could also mean that for depression and perhaps by extension tinnitus-increased anatomical precision may not be a necessary for improving efficacy and therefore not a useful parameter to optimise further. Rather than a one-size-fits-all approach based on 10–20 coordinates, computational modelling using patient-specific morphological data to ensure a sufficiently strong and correctly distributed electric field might be a more pragmatic way to ensure sufficient stimulation of the intended cortical target(s).

The second parameter for which network meta-analysis was possible was the number of tDCS sessions. Depending on resources and loss to follow-up, a potentially endless number of tDCS sessions could be applied. Evidence regarding a more specific optimal number of sessions is therefore desirable for optimisation. The number of sessions deemed most likely to be optimal by network meta-analysis was seven. Since there was only one study included that administered seven sessions of tDCS, this option may have been given undue weight in the model. However, on closer examination, both five and 10 sessions also ranked highly, with both options supported by multiple studies. This seems to indicate that the optimal number of sessions is somewhere between five and 10, perhaps at seven or very close. This could guide future efficacy trials. However, if a more specific number of sessions is required, this effect may need to be further investigated in a dose-response trial.

The final parameter for which network meta-analysis was possible was session duration. The network meta-analysis showed that 20 min was likely to be optimal. This was the most frequently reported session duration. The ranking of the other reported session durations suggests that shorter durations are less likely to be optimal and that more is unlikely to be gained from a longer duration (30 min).

While the network meta-analyses shed some light on the relationship between variations in stimulation parameters and treatment outcome of tDCS in depression, it must be noted that there is a lot of uncertainty around the estimates these analyses produced. Given the relationship between the effects of tDCS on tinnitus and depression, the parameters likely to be effective for depression, could have guided optimisation efforts of stimulation parameters for tinnitus. However, given the uncertainty around the probabilities resulting from the meta-analyses in this review, this is not reasonable. These results highlight the need for more high-quality data both for depression, and tinnitus, and anxiety for which data was not sufficient to even attempt this type of analysis.

The meta-analysis for anxiety did not result in a statistically significant effect, though a trend in the same direction in favour of active tDCS. Given the diagnostic overlap between depression and anxiety [68], one would normally expect similar results for these outcomes. While symptoms of depression and anxiety were measured in many included studies, RCTs involving participants with a diagnosis of any health condition were included, meaning many participants did not have a diagnosis of clinical depression or an anxiety disorder. There was also limited data available in comparison to the depression data, meaning insufficient data may have been available to demonstrate an effect.

Several limitations must be acknowledged, in the first place relating to the included evidence. Including studies with a population with any health condition allowed for the capture of previously untapped data when investigating the effect of tDCS on tinnitus and co-occurring depression and anxiety. It is however a source of heterogeneity which, though statistically accounted for, may have affected the result. Furthermore, studies were grouped by different options in the same parameter but differed on other parameters. This leads to a multiplication of differences in study design. For tinnitus, and perhaps anxiety, insufficient data is currently available to allow for optimisation of tDCS protocols for tinnitus, hindering the progress in this field.

Further limitations relating to the process of this review must also be mentioned. By excluding studies that used HD-tDCS, heterogeneity was reduced. This does, however, mean that no conclusions regarding HD-tDCS can be drawn from this data. While the network meta-analyses were able to shed light on the roles of stimulation parameters in the effect of tDCS on depression, it must be emphasised that these analyses provide probabilities with wide confidence intervals, and all relevant statistical reservations apply.

## 5. Conclusions

This systematic review sought to assess the effects of tDCS on tinnitus symptom severity, depression, and anxiety, as well as the relationship between stimulation parameters and these effects. By synthesising the data from RCTs regarding tDCS for tinnitus, depression, and anxiety, it has provided a robust overview of the existing evidence base. The meta-analyses have shown a statistically significant effect of tDCS, compared to sham, on both tinnitus and depression with moderate and low certainty respectively, but not anxiety. The network meta-analyses on the depression data have given an indication for possible optimal parameters. The results of the results of the meta-analyses of tDCS session duration and session length provide opportunities for further optimisation of this intervention. The results of the network meta-analysis on electrode montage have shown that, at least for conventional tDCS, spatial specificity may not be an important factor in treatment effectiveness and may therefore not be a suitable candidate parameter for further optimisation. These findings will inform the design and conduct of future efficacy trials of tDCS for tinnitus.

## Figures and Tables

**Table 1:** Study characteristics.

Reference	Type of Study	Condition Studied	Primary Outcomes Measured	Instrument	Sample Size	Electrode Montage	Current Intensity	Session Duration	Number of Sessions
Acler et al., (2013) [34]	Parallel RCT	Post-Polio Syndrome	Depression	HDRS	32	two anode 2 cm ahead of C3 and C4, cathode over left shoulder	1.5 mA	15 min	15
Ahmadizadeh et al., (2019) [35]	Parallel RCT	PTSD	Depression, anxiety	BDI-II, BAI	34	anode over F3, cathode over F4;	2 mA	20 min	10
Azmoodeh et al., (2021) [36]	Parallel RCT	Epilepsy	Depression, anxiety	DASS-21	30	anode over F3, cathode over F4;	1.5 mA	20 min	10
Benninger et al., (2010) [37]	Parallel RCT	Parkinson’s Disease	Depression	BDI	25	anode 10 mm anterior to Cz, two 25 cathodes over mastoids	2 mA	20 min	8
Bimorgh et al., (2020) [38]	Parallel RCT	Opioid dependence	Depression, anxiety	DASS-21	27	anode over F4, cathode over F3	2 mA	20 min	7
Blumberger et al., (2012) [39]	Parallel RCT	Depression	Depression	MADRS, HDRS-17, BDI-II	24	anode over F3, cathode over F4	2 mA	20 min	15
Caumo et al., (2021) [40]	Parallel RCT	Fibromyalgia	Depression	BDI-II	32	anode over F3, cathode over Fpz	2 mA	20 min	20
Cavalcanti et al., (2015) [41]	Parallel RCT	Tinnitus	Tinnitus	THI	18	anode over F4, cathode over F3	2 mA	20 min	5
Dantas et al., (2020) [42]	Parallel RCT	Dysmenorrhea	Anxiety	HAS	24	anode over F3, cathode over Fp2	2 mA	20 min	5
Forogh et al., (2016) [43]	Parallel RCT	Tinnitus	Tinnitus	THI	22	anode electrode halfway between C3 and T5, cathode over Fp2	2 mA	20 min	5
Fregni et al., (2006) [44]	Parallel RCT	Central pain following spinal injury	Depression	BDI	17	anode electrode over C3 or C4, cathode electrode over the contralateral supraorbital area	12 mA	20 min	5
Grazzi et al., (2020) [45]	Parallel RCT	Chronic migraine with medication overuse	Depression, anxiety	BDI, STAIT	135	anode over C4, cathode over contralateral supraorbital area or cathode over C4, anode over contralateral supraorbital area	2 mA	20 min	5
Ibrahim et al., (2018) [46]	Parallel RCT	Visceral pain in hepatocellular carninoma	Depression	HDRS	48	anode over the primary motor cortex of the contralateral hemisphere of the most painful abdominal area was used to determine the primary motor area (M1) of the patient, cathode over the opposite supraorbital region cathode F3, anode over F4	2 mA	30 min	10
Jafari et al., (2021) [47]	Parallel RCT	Social anxiety disorder	Depression	BDI-II	24	anode over F3, cathode over F4;	1 or 2 mA	20 min	10
Klauss et al., (2014) [48]	Parallel RCT	Alcohol dependance	Depression, anxiety	HDRS, HARS	35	cathode over F3, anode over F4	2 mA	13 min	5
Liu et al., (2016) [49]	Parallel RCT	Temporal lobe epilepsy	Depression	BDI	33	anode over F3, cathode over Fp2	2 mA	30 min	5
Loo et al., (2010) [50]	Parallel RCT	Depression	Depression	MADRS	40	anode over the left dorsolateral prefrontal cortex (DLPFC), identified as pF3	1 mA	20 min	5
Loo et al., (2012) [51]	Parallel RCT	Depression	Depression	MADRS	64	anode over pF3, cathode over F8	2 mA	20 min	15
Loo et al., (2018) [52]	Parallel RCT	Depression	Depression	MADRS	84	anode over F3, Cathode over F8	2.5 mA	30 min	20
Mariano et al., (2018) [53]	Parallel RCT	Chronic lower back pain	Anxiety	GADS	21	cathode over FC1, anode over contralateral (right) mastoid	2 mA	30 min	10
Movahed et al., (2017) [54]	Parallel RCT	Anxiety	Depression, anxiety	HDRS, HARS	12	anode on the left deltoid and cathode over F4	2 mA	20 min	10
Natividade et al., (2019) [55]	Parallel RCT	Obesity	Depression	BDI	28	anode electrode over F4, cathode over F3	2 mA	20 min	20
Pal et al., (2015) [56]	Parallel RCT	Tinnitus	Tinnitus, depression, anxiety	THI, HADS	42	anode at F3-Fz-F4 and two cathodes 35.75 cm^2^ each at T3	2 mA	20 min	5
Palm et al., (2013) [57]	Crossover RCT	Depression	Depression	HDRS	22	anode over F3, cathode over Fp2	1 or 2 mA	20 min	10
Pegado et al., (2019) [58]	Parallel RCT	Dysmenorrhea	Anxiety	HARS	22	anode over C3, cathode over Fp2	Not reported	20 min	5
Salehinjad et al., (2017) [59]	Parallel RCT	Depression	Depression	BDI	24	anode over F3, cathode over F4	2 mA	20 min	10
Samartin-Veiga et al., (2021) [60]	Parallel RCT	Fibromyalgia	Depression	HADS	54	C3 and Fp2 or F3 and Fp2	2 mA	20 min	15
Sampaio-Junior et al., (2017)	Parallel RCT	Bipolar depression	Depression	HDRS, MADRS	59	anode right dlPFC, cathode left dlPF	2 mA	30 min	12
Sharafi et al., (2019) [61]	Parallel RCT	Depression	Depression	HDRS	30	anode F3 cathode F4	2 mA	20 min	10
Souza et al., (2020) [62]	Parallel RCT	Tinnitus	Tinnitus	THI	24	anode over CP5, cathode over F4	2 mA	20 min	5
Valiengo et al., (2016) [63]	Parallel RCT	Post-stroke depression	Depression	HDRS, MADRS	48	anode over F3, cathode over F4	2 mA	30 min	12
Vigod et al., (2019) [64]	Parallel RCT	Antenatal depression	Depression	MADRS, Edinburgh Postnatal Depression Scale	20	anode over F3, cathode over F4	2 mA	30 min	15
Yadollahpour et al., (2017) [24]	Parallel RCT	Tinnitus	Tinnitus, depression, anxiety	THI, BDI-II, BAI	42	anode over F4, cathode over F3	2 mA	20 min	5
Yadollahpour et al., (2018) [65]	Parallel RCT	Tinnitus	Tinnitus	THI	40	anode halfway between T3 and F7, halfway between T4–F8	2 mA	20 min	10
Young et al., (2021) [66]	Parallel RCT	Chronic neuropathic pain in MS	Depression, anxiety	DASS-21	30	anodal electrode applied to the C3 or C4 contralateral to the side of pain; if both sides were affected, the side with higher pain level was selected, cathode over the supraorbital area contralateral to the stimulated motor cortex	2 mA	20 min	5
Zhou et al., (2020) [67]	Parallel RCT	Insomnia	Depression	SDS	90	anode over left DLPFC and cathode over right DLPFC	2 mA	30 min	24

**Table 2:** Results: network meta-analysis electrode montage.

	A-F3/C-F4		A-F3/C-Fp2		A-F4/C-F3		Sham	
	Probability	95% CI	Probability	95% CI	Probability	95% CI	Probability	95% CI
Best	38.9	−0.591, 1.369	38	−0.6, 1.36	23.2	−0.552, 1.016	0	0
Second	40	−0.58, 1.38	26.1	−0.523, 1.045	33.9	−0.641, 1.319	0	0
Third	21.1	−0.573, 0.995	33	−0.65, 1.31	42.1	−0.559, 1.401	3.8	−0.354, 0.43
Worst	0	0	2.9	−0.363, 0.421	0.9	−0.106, 0.286	96.2	0.57, 1.354
Mean rank	1.8	0	2	0	2.2	0	4	0

**Table 3:** Results: network meta-analysis number of sessions.

	5 Sessions		7 Sessions		8 Sessions		10 Sessions		12 Sessions
	Probability	95% CI	Probability	95% CI	Probability	95% CI	Probability	95% CI	Probability
Best	0	0	89.3	0.305, 1.481	1.1	−0.185, 0.207	5.5	−0.337, 0.447	1.4
Second	0.2	0.02, 0.02	6.7	−0.521, 0.655	6.5	−0.327, 0.457	61.2	−0.368, 1.592	7.9
Third	6.5	−0.327, 0.457	2.2	−0.174, 0.218	10.9	−0.479, 0.697	27.2	−0.512, 1.056	14.1
Fourth	18.8	−0.596, 0.972	1.1	−0.185, 0.207	12.9	−0.459, 0.717	5.4	−0.338, 0.446	14.8
Fifth	27.6	−0.508, 1.06	0.4	−0.192, 0.2	11.1	−0.477, 0.699	0.6	−0.19, 0.202	11.7
Sixth	23.7	−0.547, 1.021	0.2	0.002, 0.002	10.1	−0.487, 0.684	0.1	0.001, 0.001	10.2
Seventh	14.5	−0.639, 0.929	0.1	0.001, 0.001	9.6	−0.492, 0.684	0	0	8.8
Eighth	6.5	−0.327, 0.457	0.1	0.001, 0.001	11	−0.478, 0.698	0	0	10.5
Worst	2	−0.176, 0.216	0	0	26.9	−0.515, 1.053	0	0	20.6
Mean rank	5.5	0	1.2	0	6.1	0	2.3	0	5.7
		**15 Sessions**		**20 Sessions**		**24 Sessions**		**Sham**	
	**95% CI**	**Probability**	**95% CI**	**Probability**	**95% CI**	**Probability**	**95% CI**	**Probability**	**95% CI**
Best	−0.182, 0.21	0	0	0	0	2.7	0.027, 0.027	0	0
Second	−0.509, 0.667	0.5	−0.191, 0.201	1.1	−0.185, 0.207	15.9	−0.625, 0.943	0	0
Third	−0.447, 0.729	6.7	−0.521, 0.655	6.9	−0.519, 0.657	25.4	−0.53, 1.038	0	0
Fourth	−0.44, 0.736	15.9	−0.625, 0.943	12.2	−0.466, 0.71	18.8	−0.596, 0.972	0.1	0.001, 0.001
Fifth	−0.471, 0.705	20.3	−0.581, 0.987	15.6	−0.628, 0.94	11	−0.478, 0.698	1.6	−0.18, 0.212
Sixth	−0.486, 0.69	20.7	−0.577, 0.991	16.9	−0.615, 0.953	8	−0.508, 0.668	10.1	−0.487, 0.689
Seventh	−0.5, 0.676	16.7	−0.617, 0.951	15.7	−0.627, 0.941	5.9	0.333, 0.451	28.8	0.692, 1.268
Eighth	−0.483, 0.693	11.9	−0.469, 0.707	15.5	−0629, 0.939	6	−0.332, 0.452	38.5	−0.595, 1.365
Worst	−0.578, 0.99	7.1	−0.517, 0.659	16.2	−0.622, 0.946	6.3	−0.329, 0.455	20.9	−0.575, 0.993
Mean rank	0	5.9	0	6.3	0	4.4	0	7.7	0

**Table 4:** Results: network meta-analysis session duration.

	10 min		13 min		15 min		20 min		30 min		Sham	
	Probability	95% CI	Probability	95% CI	Probability	95% CI	Probability	95% CI	Probability	95% CI	Probability	95% CI
Best	16.1	−0.623, 0.945	2.5	−0.367, 0.417	10.5	−0.483, 0.693	58.6	−0.394, 1.566	12.3	−0.465, 0.711	0	0
Second	10.3	−0.485, 0.691	3.6	−0.356, 0.428	9.6	−0.492, 0.684	32.3	−0.657, 1.303	44	−0.54, 1.42	0.3	−0.193, 0.199
Third	14.4	−0.64, 0.928	9.6	−0.492, 0.684	17	−0.614, 0.954	8.1	−0.507, 0.669	33.1	−0.649, 1.311	17.9	−0.605, 0.963
Fourth	14.2	−0.446, 0.73	13.6	−0.452, 0.724	18.6	−0.598, 0.97	1.1	−0.185, 0.207	9.3	−0.495, 0.681	43.2	−0.548, 1.412
Fifth	18.7	−0.597, 0.971	26.4	−0.52, 1.048	22.1	−0.563, 1.005	0	0	1.2	−0.184, 0.208	31.6	−0.664, 1.296
Worst	26.3	−0.521, 1.047	44.4	−0.536, 1.424	22.2	−0.562, 1.006	0	0	0.2	0.002, 0.002	7	−0.518, 0.658
Mean rank	3.9	0	4.9	0	4	0	1.5	0	2.4	0	4.3	0

**Table 5:** GRADE assessment tinnitus.

Certainty Assessment							№ of Patients		Effect		Certainty
№ of Studies	Study Design	Risk of Bias	Inconsistency	Indirectness	Imprecision	Other Considerations	Active	Sham Tinnitus	Relative (95% CI)	Absolute (95% CI)	
6	randomised trials	serious ^a^	serious ^b^	not serious	not serious	strong association	103	85	-	MD 11.62 lower (18.94 lower to 4.31 lower)	⨁⨁⨁◯ Moderate

**CI:** confidence interval; **MD:** mean difference. Explanations: ^a^ The two most heavily weighted studies were both deemed to have a high risk of attrition bias. ^b^ I2 = 49% indicating a medium degree of inconsistency.

**Table 6:** GRADE assessment depression.

Certainty Assessment							№ of Patients		Effect		Certainty
№ of Studies	Study Design	Risk of Bias	Inconsistency	Indirectness	Imprecision	Other Considerations	Active	Sham Depression	Relative (95% CI)	Absolute (95% CI)	
30	randomised trials	serious ^a^	very serious ^b^	not serious	not serious	strong association	644	562	-	SMD 0.61 lower (0.86 lower to 0.35 lower)	⨁⨁◯◯ Low

**CI:** confidence interval; **SMD:** standardised mean difference. Explanations: ^a^ 11 studies were deemed to have a high risk of bias in at least one domain. ^b^ I2 = 76% indicating a high degree of inconsistency.

**Table 7:** GRADE assessment anxiety.

Certainty Assessment							№ of Patients		Effect		Certainty
№ of Studies	Study Design	Risk of Bias	Inconsistency	Indirectness	Imprecision	Other Considerations	Active	Sham Anxiety	Relative (95% CI)	Absolute (95% CI)	
15	randomised trials	serious ^a^	very serious ^b^	not serious	serious ^c^	none	326	266	-	SMD 0.28 lower (0.93 lower to 0.37 higher)	⨁◯◯◯ Very low

**CI:** confidence interval; **SMD:** standardised mean difference. Explanations: ^a^ 5 studies were deemed to have high risk of bias in at least one domain. ^b^ I2 = 92% indicating a high degree of inconsistency ^c^ Wide confidence intervals overlapping line of the null effect.

## Supplementary Materials

The following supporting information can be downloaded at: https://www.mdpi.com/article/10.3390/brainsci12040484/s1. Table S1: Risk of bias.

## PROSPERO Registration Number

CRD42020185567—https://www.crd.york.ac.uk/prospero/display_record.php?RecordID=185567 (accessed on 5 April 2022).
