# Supplementary figures and images for: Determining the Effects of Transcranial Direct Current Stimulation on Tinnitus, Depression, and Anxiety: A Systematic Review
Source: Brain Sci. 2022 Apr 8;12(4):484. doi: 10.3390/brainsci12040484 (PMC9029345; doi:10.3390/brainsci12040484)

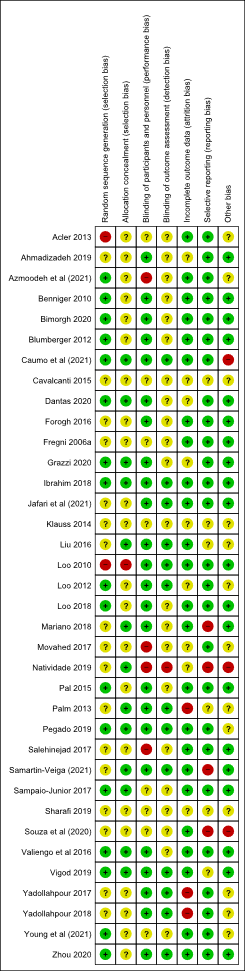

Supplement: Supplementary file 1 [file brainsci-12-00484-s001.zip › Supplementary Table 1.png]
